# Exploring factors that contribute to the successful implementation of Schwartz Rounds in higher education institutions

**DOI:** 10.1186/s12909-026-08793-9

**Published:** 2026-02-11

**Authors:** Sarah Beck, Cath Taylor, Jill Maben

**Affiliations:** Faculty of Health and Medical Sciences, School of Health Sciences, University of Surrey, Guildford, UK

**Keywords:** Schwartz rounds, Student wellbeing, Higher education, Implementation

## Abstract

**Background:**

With rising concerns about workforce shortages and early-career attrition, there is increasing focus on the difficulties healthcare students encounter during training. Schwartz Rounds are a structured group intervention where healthcare staff share stories about the emotional, ethical and social impact of their work. As students spend substantial time in clinical settings, Higher Education Institutions (HEIs) are adopting Rounds to support wellbeing and foster compassionate care. While evidence suggests that Rounds are well received by students, less is known about how to ensure successful implementation in this setting.

**Methods:**

Longitudinal, mixed-methods (qualitative dominant) case studies were conducted in six HEIs in the South of England between April 2022-December 2024. Data collection across 20 months included semi-structured interviews with those running and attending Rounds (*n*=19), non-participant observations of Rounds activities (including steering group meetings, storyteller preparation meetings, and Rounds) (*n*=38), post-Round feedback surveys (*n*=481), and bespoke forms with key information about each Round (e.g., attendance) (*n*=32). Quantitative data were analysed descriptively, and qualitative data thematically. Data were summarized in matrices to examine patterns within and across sites using the Framework Method and mapped to the Consolidated Framework of Implementation Research.

**Results:**

Five of the six HEIs implemented Rounds during the study period. Key drivers included support (financial, resources) and credibility from external organisations. Within the HEIs, four interlinked features of the inner-setting and intervention aided successful implementation: 1) Motivation of Schwartz team, with facilitators championing Rounds, senior clinical leads lending authority, active steering groups, and tailored administrative support); 2) Engagement activities to inform students what Rounds are and when/where they took place; 3) Involving students in delivery to foster ownership; 4) Engaging wider staff to increase awareness and endorsement. These activities increased familiarity with Rounds over time and produced early signs of them becoming embedded in university culture.

**Conclusions:**

This study offers new insights into the implementation of Schwartz Rounds in HEIs, highlighting key enablers and barriers. Success was shaped by local adaptation, leadership engagement, team support and student involvement. Findings can aid adoption and implementation of Schwartz Rounds in HEIs and other organisations aiming to support student practice and wellbeing.

**Supplementary Information:**

The online version contains supplementary material available at 10.1186/s12909-026-08793-9.

## Background

Amid global concerns over healthcare workforce shortages and high rates of early-career attrition [1, 2], increasing attention has turned to the challenges healthcare students face during their training. Alongside intensive academic demands [3], students spend significant time in clinical placements exposing them to the complexities of practice [4]. Students navigate discrepancies between expectations and reality of the profession [5], worries about competence [6], and emotional demands from patients and colleagues [7]. These experiences can contribute to high levels of stress and low wellbeing [3, 7, 8], high course attrition [5, 9], and reduced quality and compassion of care [10, 11, 12]. Consequently, there is growing consensus that higher education institutions must strengthen support systems and cultivate compassionate learning environments [13, 14, 15].

### Schwartz Rounds

Schwartz Rounds (Rounds) are a structured, group intervention designed to support healthcare staff by fostering reflection and compassion. Rounds were inspired by Kenneth Schwartz, who while terminally ill, recognised how small acts of kindness by staff made “the unbearable, bearable” [16], but for staff to provide compassionate care they needed spaces to share and reflect on the impact of caring on them. First established in the US by the Schwartz Center for Compassionate Care, Rounds were brought to the UK in 2009 by the Point of Care Foundation (PoCF) who oversee licensing, training and mentorship.

In Rounds, one or more staff members share their stories on an agreed theme or anonymised patient case (supported by trained Rounds facilitators in a preparatory session beforehand) regarding the emotional, ethical and social impact of work [17]. Examples of stories include ‘A patient I’ll never forget’, ‘The day I made a difference’, and ‘Lost in translation’. The stories are intended as a vehicle to resonance and reflection by staff in the audience, and facilitators will encourage sharing of those reflections and second stories. Rounds are held in person or online, and follow a set format designed to prioritise confidentiality and psychological safety. After providing space and time to unwind from work, Rounds start with an introduction by facilitators to explain what Rounds are (and not) and how the session will run before the storytellers are invited to share their stories [17]. Facilitators then guide group reflection, encouraging audience members to share if they wish, while holding silences and avoiding problem solving. Storytellers then offer final reflections before the session ends on time, usually after sixty minutes.

Running Rounds requires a clinical lead, facilitators, a steering group and administrative support. Clinical leads, often senior staff, symbolise institutional support and may co-facilitate. Facilitators source and prepare storytellers and lead Rounds. The steering group reflect on past Rounds, support facilitators, review evaluations, help promote Rounds, identify future themes and source storytellers. Administrators manage logistics, meetings (e.g. for steering group, storyteller preparation), communications, food provision if face-to-face and feedback collection.

Unlike other interventions, Rounds provide an ongoing, organisation-wide forum [19]. A UK national evaluation of Rounds in healthcare settings [17, 20] found regular attendance was associated with higher empathy, greater compassion for self and others, lower psychological distress, and that they created a space to reflect on work challenges – improving teamwork and promoting positive cultural change.

### Rounds in Higher Education Institutions (HEIs)

Since 2015 the PoCF has supported the implementation of Rounds within HEIs. Here, Rounds stories tend to be shared on an agreed theme rather than specific patient cases and are usually limited to health or social care programmes. Students have responded positively to Rounds, noting enhanced professional identity, emotional validation, and desire to continue attending [21, 22, 23]. Reviews highlight Rounds as safe spaces where students can share emotions, reduce isolation, observe role-modelling of vulnerability and build confidence and interpersonal connections [4, 24].

However, current evidence is of mixed quality and is predominately single case, cross-sectional student evaluations or discussion articles [4, 24]. It is noted that power dynamics between students and educators in HEIs [24] may impact Rounds’ implementation and sustainability.

In 2022, building on the success of Schwartz North [25], Health Education England (HEE) commissioned the launch and evaluation of ‘Schwartz South’, supporting HEIs in the South of England to implement Rounds. This provided an opportunity to examine implementation of Rounds across a range of HEIs and thereby contribute to the growing evidence base on Rounds in this setting.

### Aim

To identify the key drivers to successful implementation of Rounds within HEIs, using Schwartz South as an exemplar.

## Methods

### Design

A mixed-methods (qualitative dominant) longitudinal case study design was used to evaluate implementation of Rounds across six HEIs [26]. A case-study design was considered appropriate as it allowed in-depth exploration of how Rounds were implemented and experiences within different institutional contexts over time, while also allowing for comparison across sites. Successful implementation was defined as delivery of Rounds with high fidelity to the PoCF Schwartz Rounds model, frequent and successful Rounds (good uptake, acceptability and regularity), and evidence over time of integration into organisational strategy and practices (e.g. pathway to normalisation [27]).

Schwartz South was designed as a Hub-and-Spoke model, with the University of Surrey (the hub) supporting HEIs (spokes) to implement Rounds. Drawing on its experience delivering and evaluating Rounds [17, 28], the University of Surrey, shared resources, and offered ongoing formal (three online events) and ad-hoc support throughout the project period, culminating in a celebration event (April 2022-December 2024). Each spoke site received funding to cover the PoCF licence fee (for the first 2 years), including access to training, support and resources from the PoCF, and a contribution towards administrator/running costs.

### Sampling of participant sites

Advertisements were sent to all HEIs with healthcare programmes in the South of England inviting expressions of interest to join Schwartz South. Recruitment occurred in two waves: May 2022 (Cohort one) and September 2022 (Cohort two). Eight expressions of interest were received; two were not eligible due to either already having Rounds established or being out of region. Six HEIs joined Schwartz South and participated in this study, with three in each Cohort.

### Data collection

Data were collected from November 2022–July 2024. Data collection was aimed at enabling longitudinal investigation of all four stages of Rounds [17]: (1) sourcing stories/storytellers, (2) preparing storytellers, (3) the Round itself, and (4) after-effects/impact of Rounds. Data collection methods included (a) bespoke data collection forms; (b) a post-Rounds attendance questionnaire; (c) semi-structured interviews; (d) non-participant observations of Rounds, storyteller preparation and steering group meetings.

#### Bespoke data collection forms

Forms were completed by the clinical lead, facilitator or administrator to collect data for each Round on title; date and time; number of students registered to attend; titles of courses/programmes invited to attend; total number of attendees and storytellers (including whether staff or students).

#### Post-rounds attendance questionnaire

A feedback questionnaire was distributed to all participants post-attendance either in hardcopy or shared digitally via Qualtrics (via a QR code or anonymised link). The questionnaire included items previously used in the national evaluation of Rounds [17], and items adapted from the post-Round survey developed by the PoCF [18]. A copy of the questionnaire can be found in Supplementary material 1. Due to the focus on evaluating implementation, three items were selected for analyses here: the overall Round rating (from poor to exceptional); and factors that enabled and prevented attendance (multiple choice with additional free-text option).

#### Semi-structured interviews

Topic guides, informed by Rounds implementation evidence [17, 29], were tailored to the interviewee role. Questions explored participants’ experiences of Rounds, including barriers and facilitators to implementation and engagement. We aimed to interview at least one facilitator, clinical lead, steering group member, administrator, attendee and storyteller at each site. Participants were invited (by email or in person) after their site had held at least two Rounds. Interviews were scheduled throughout the study period to gather data on experiences over time. Interviews were conducted individually via Microsoft Teams, were audio recorded, and lasted 38–84 min (M = 55 min).

#### Non-participant observations

Observational fieldnotes from Rounds, storyteller preparation and steering group meetings were captured using bespoke data collection forms. Forms were adapted from a previous study [29] (see Supplementary material 2) and allowed for both inductive and deductive data collection. Data collected are summarised in Table 1.

**Table 1:** Observational data collection

Observation form	Contextual data	Key elements to ensure safety & fidelity (taken from PoCF training manual)	Other observations
Rounds	• Day and time • Theme of Round • No. facilitators • No. storytellers • No. in audience • Timings of start, introduction, stories, discussion and end • How many speaking audience members (or in the chat if online) • Roles of team members • Types/topics of stories shared (by storytellers and audience)	Comments on the following: • Introduction to Round protocol • Boundaries for safety • Sensitive space • Time management • Managing silence and speaking • Avoiding problem solving • Drawing out underlying meaning of stories • Maintaining neutral curiosity	• Any issues experienced • Audience contributions • Management of emotions and interpersonal dynamics • How the Round was ended
Steering group meetings	• Day and time • Agenda and minutes taken • Chairing/leadership of meetings • Attendance (of staff and student representatives) • Meeting structure • Content of discussion	• Time management • Opportunity and receptiveness for sharing views • Feedback from previous Rounds • Planning for future Rounds	• Atmosphere and interpersonal dynamics • Sourcing of storytellers
Storyteller preparation	• Day and time • How many participants • Theme • Structure of session • Topic and structure of stories	• Introduction by facilitators • The emotional/relational content drawn out (with similarities and differences between stories) • Facilitators management of readiness and emotion in telling story • Facilitator setting boundaries for safety • Understanding of storyteller of Rounds purpose and what is required	• Time of storyteller to reflect on own story • How meeting ended • Notes on story preparation

### Data analysis

The framework method [30] was used to integrate and analyse interview, observation and survey data within and between each case study site (Table 2). This allowed exploration of within-site patterns and cross-site comparisons, while considering their individual context. Students’ Round ratings from the survey were initially included, but the limited variation between sites (all highly rated, Range M = 4.0 (SD = 0.0)-4.4 (SD = 0.6)) and variable sample sizes (2-318) meant this data did not help to explore variation between Rounds or sites, and this data was therefore excluded.

Data were analysed both inductively and deductively when identifying codes, followed by a further deductive analysis using an analytical framework informed by the Consolidated Framework for Implementation Research (CFIR) [31], Table 2). The CFIR provides a comprehensive theoretical framework of the determinants of implementation, aiding evaluation of health-related interventions. It includes features of the intervention itself, the inner setting (or organisational context), as well as the outer setting (e.g., wider HEI network, NHS). Through synthesis across data sources and sites and charting barriers/enablers into a matrix, key drivers to implementation were identified.

**Table 2:** Stages of analysis using the framework method [30]

Stage of analysis	Analysis procedures
Stage 1: Transcription/ Data preparation	Audio recordings of interviews were transcribed verbatim and handwritten observation forms were typed into Microsoft Word. Survey data were downloaded into SPSS (Version 29) for frequency analysis of multiple-choice responses regarding barriers and enablers to attendance. Free-text responses were analysed where responses were either categorised into existing themes (from the multiple-choice options) or used to create new themes.
Stage 2: Familiarisation	All data was organised per site and reviewed (read and re-read) to familiarise with initial topics and narratives. During this stage, the research team planned the strategy for analysis and discussed initial comments from familiarisation.
Stage 3: Initial Coding	Initial coding was completed for two sites. Interviews were analysed first, inductively, to identify enablers and barriers of implementation. Next, observation forms were analysed inductively to search for additional codes, as well as deductively searching for features that would support, or conflict, with those already identified in interviews. Finally, survey data was reviewed to provide additional student perspectives on engagement.
Stage 4: Developing analytic framework	The research team met to discuss emerging codes and their inter-relations. The relevance and usefulness of using an existing evidence-based implementation framework to code and organise data was discussed and agreed (CFIR) [31]. Based on this, the analytic framework was created whereby data were also coded according to domains (and constructs) within the CFIR (including contextual factors of each HEI).
Stage 5: Applying the framework	All data was then coded according to this framework for each site. Emerging findings were discussed in regular research team meetings leading to further analysis and development of codes.
Stage 6: Charting to framework matrix	Key barriers and facilitators to implementation were then charted into large matrices for each domain of the CFIR, including contextual data for each site to enable exploration of patterns within and between sites.
Stage 7: Interpreting the data	Patterns within and between sites of barriers and enablers to implementation under the CFIR constructs were identified and analysed by the team resulting in identification of the key determinants of successful implementation of Rounds.

### Ethical considerations

The study was approved by the University of Surrey ethics committee (FHMS 22–23 001). Rounds attendees received study information and provided consent upon registration. At the Round attendees were reminded of the study, that the Round was being observed and were given the opportunity to leave prior to the Round starting. Informed consent was obtained before questionnaires, interviews and non-participant observations. Questionnaires were anonymous, and sites were given pseudonyms during data collection and analysis to ensure anonymity. Quotations are attributed only to the role of the interviewee. Cohort One (Peony, Daisy and Cornflower) began in 2022 and Cohort Two (Rose, Lavender and Daffodil) in 2023.

## Results

### Characteristics of sites

Six HEIs in the South of England joined the Schwartz South project as case study sites. There was variation across sites in relation to the number of total enrolled students (7,000–35,000), health/social care students (200-2,000) and types of health/social care courses ran (Table 3). Rounds were run across two or more sites in three of the HEIs.

**Table 3:** Characteristics of sites

Site	HEI size (Number of students)*	Number of Healthcare / social care students* (Sites based across)	Courses invited to Rounds
Peony	17,000	800 (2 sites)	Adult Nursing, Child Nursing, Mental Health Nursing, Physiotherapy, Occupational Therapy, Podiatry, Paramedic Science, Midwifery, Health And Social Care, Diagnostic Radiography
Daisy	8,000	700 (1 site)	Nursing, Physiotherapy, Social Work, Nutrition And Dietetics, Occupational Therapy
Cornflower	17,000	700 (3 sites)	Adult Nursing, Children And Young People Nursing, Midwifery, Mental Health Nursing, Physiotherapy, Occupational Therapy, Operating Department Practice, Paramedic Science, Social Work
Rose	7,000	350 (1 site)	Nursing, Physiotherapy, Social Work
Lavender	35,000	2,000 (1 site)	Nursing, Midwifery, Radiography, Paramedic Science, Dietetics
Daffodil	30,000	200 (2 sites)	Adult Nursing

* Rounded to nearest 100 to preserve anonymity

### Rounds conducted and data collected

Of the six HEIs, five implemented Rounds during the data collection period. Daffodil did not (with reasons explored throughout the results section). Of those that implemented Rounds, the number of Rounds run varied (3–11), whether in-person or online, as did average student attendance (13–77, Table 4). All five sites invited steering group members (e.g. staff) to attend Rounds, and three sites (Peony, Daisy and Rose) also included staff members as storytellers. Observational data were collected in 14 Rounds (2–4 per site), 14 steering group meetings (2–4) and ten storyteller preparation meetings (1–4). Nineteen interviews (2–5 per site) were conducted (Role information of those interviewed is presented in Table 4), and 481 survey responses were received (2-318, Table 4).

**Table 4:** Rounds conducted and data collected in each case study site

		Rounds conducted					Data collected				
	Site	Rounds *N*	*N* In person (online)	Mean sign up(SD)	Mean student attendance (SD, Range)	Range of no. storytellers	Rounds observed *N*	Steering group meetings observed *N*	Storyteller preparation observed *N*	Interviews conducted *N* (Roles interviewed)	Survey responses *N* (Response rate %)
Cohort one	Peony	11	8(3)	69.3(32.6)	36.3 (21.5, 10–82)	1–4	4	2	2	4 (F, CL, Ad, A)	318 (79.6)
	Daisy	9	9(0)	31.4(18.4)	22.6 (15.3, 5–49)	2–4	2	4	4	5 (F, SGM, Ad, 2As)	34 (46.6)
	Cornflower	5	3(2)	29.0(21.2)	14.6 (21.2, 1–52)	1–2	4	4	2	4 (F, CL, Ad, ST)	14 (19.2)
Cohort two	Rose	3	(3)	28.0(8.5)	13.3 (4.7, 8–17)	3	2	2	1	4 (F, SGM, Ad, A)	2 (5.0)
	Lavender	4	4	N/A*	77 (35.1, 40–116)	3–4	2	2	1	2 (1 F, 1 CL)	113 (36.7)
	Daffodil	0	-	-	-	-	-	-	-	-	-
Total		32	24 (8)	45.5(30.7)	32.0 (27.7, 1-116)	1–4	14	14	10	19	481

*Roles interviewed: F *facilitator,* CL *Clinical lead,* SGM *Steering Group Member,* Ad *Administrator,* A *Attendee,* ST *Storyteller

*Lavender timetabled Rounds (therefore students were not required to sign up in advance). Daffodil did not run any Rounds

In relation to enablers and barriers to Rounds attendance, the most common enabler across sites was the suitability of the time of day, with preferred times varying between students, with no one time ideal. Sites typically scheduled Rounds at lunchtime or the end of the day. This worked for some but barriers to attendance included difficulty in sparing the time, uncertainty about what was involved, and the timing of the Round being unsuitable (Table 5 presents most frequently reported barriers/enablers; See Supplementary Table 1 for all). In contrast, one site timetabled Rounds into student modules. 

**Table 5:** Most frequent enablers and barriers to student attendance reported in survey

Total *N* Responses:	Peony % (*N*)	Daisy % (*N*)	Cornflower % (*N*)	Rose % (*N*)	Lavender % (*N*)^	Total % (*N*)
	318	34	14	2	113	481
*What encouraged you to attend this Round?*						
The time of day was right	55.7 (177)	73.5 (25)	57.1 (8)	100.0 (2)	32.7 (37)	51.8 (249)
I was not in placement/clinical practice	36.5 (116)	41.2 (14)	28.5 (4)	0.0 (0)	22.1 (25)	33.1 (159)
It was face-to-face and I was already in college	24.2 (77)	47.1 (16)	0.0 (0)	N/A	49.6 (56)	30.1 (149)
I saw a poster and the topic appealed to me	30.5 (97)	8.8 (3)	21.4 (3)	50.0 (1)	3.5 (4)	22.5 (108)
It was online and easy to attend	30.8 (98)	N/A	28.5 (4)	100.0 (2)	N/A	21.6 (104)
* Are there any challenges you experience in attending Rounds?*						
Difficult to spare the time	23.9 (76)	26.5 (9)	35.7 (5)	0.0 (0)	8.0 (9)	20.6 (99)
I didn’t know what was involved	14.5 (46)	32.4 (11)	7.1 (1)	0.0 (0)	25.7 (29)	18.1 (87)
I couldn’t get to college for the Round (face-to-face)	11.9 (38)	23.5 (8)	7.1 (1)	N/A	1.8 (2)	10.2 (49)
Rounds are scheduled at a bad time of day for me	9.4 (30)	23.5 (8)	14.3 (2)	50.0 (1)	3.5 (4)	9.4 (45)
Too expensive to get to college for the Round (face-to-face)	5.3 (17)	11.8 (4)	7.1 (1)	50.0 (1)	1.8 (2)	5.2 (25)

*Note that attendees could select more than one option; All Daisy and Lavender Rounds were face-to-face, all Rose Rounds were online; ^Rounds were timetabled within modules for students*

### Key drivers for successful implementation of rounds in HEIs

Successful implementation was defined as delivery of Rounds with high fidelity to the PoCF Schwartz Rounds model, frequent and successful Rounds (good uptake, acceptability and regularity), and evidence over time of integration into organisational strategy and practices (e.g. pathway to normalisation [27]). Based on previous research in healthcare settings it was determined that meeting these criteria would lead to Rounds being most likely to optimise their impact on students learning and wellbeing [20].

Key drivers of successful implementation were identified and mapped to CFIR domains and constructs (Supplementary Table 2 presents this mapping; definitions of CFIR constructs are provided in Damschroder et al. [31]). Successful implementation was found to be dependent on multiple factors, predominantly the characteristics of those delivering Rounds and the strategies used to engage students and encourage attendance. Those championing implementation were most successful when actively supported by a senior, clinical lead, an engaged steering group and an administrator to increase awareness of Rounds among students and staff.

**Fig. 1:**
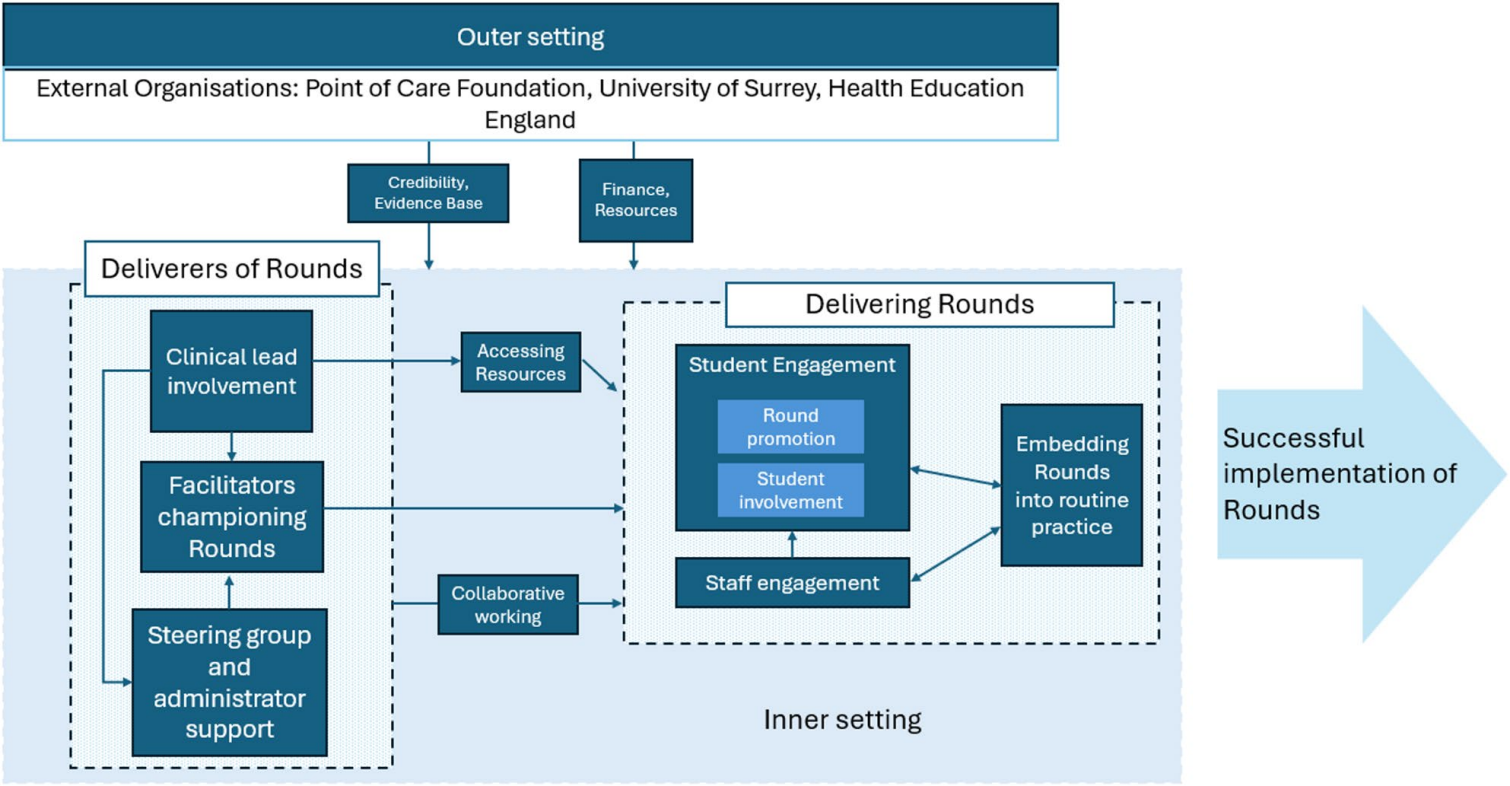
Model of successful implementation of Schwartz Rounds in HEIs

The key drivers and their relationships to each other are summarised in Fig. 1. In this model, the drivers are shown as being like a ‘dimmer switch’ to turn up the active ingredients of Rounds or not: the higher/ stronger the driver, the more successfully Rounds were implemented. Each component of the model is then described in more detail in the following sections.

### Outer setting: external organisations

External organisations (PoCF, University of Surrey and HEE) acted as the initial driver for HEIs to implement Rounds through providing formal training, financial support and sharing both knowledge and practical resources. Funding provided by HEE through the Schwartz South project enabled sites to overcome financial barriers to access training and resources needed. While sourcing additional funding continued to be a barrier to sustaining Rounds (frequently discussed in steering group meetings), the positive feedback from students obtained through being able to run Rounds (via Schwartz South) helped sites to build business cases for internal financial support beyond the project.

Partnerships with PoCF and University of Surrey (and Schwartz South) made those delivering Rounds feel part of a larger network, which became an important source of information and support, particularly in early stages of implementation:

*It feels like we are part of something bigger*,* able to ask questions*,* if we are struggling with anything*,* having the (PoCF) mentor has been a brilliant part of that process (Clinical Lead – Peony)*.

Through these connections, sites gained knowledge on Rounds and resources such as such as scripts and information packs from the University of Surrey and PoCF:

*Everything that I learned about what the Rounds actually were came from Surrey… I was sent loads of PDFs and things that were really useful (Administrator – Daisy)*.

External organisations also influenced the implementation of Rounds through providing evidence of the credibility of the intervention. Academics at the University of Surrey (the hub) led the national evaluation of Rounds in practice settings and had been running Rounds for students since 2019. They therefore were considered reputable and provided the evidence base for justifying investment and implementation of the intervention to their organisation. This context encouraged organisations to join Schwartz South, and gave confidence to those delivering Rounds when encouraging colleagues and students to consider being involved:

*I was evangelising to people about Rounds since day one. I felt that the Surrey project gave me more credibility… It wasn’t just my idea of something*,* I sent the paper – see*,* it’s evidence-based (Facilitator – Daisy)*.

### Inner setting: deliverers of rounds

Within each HEI, the level of involvement and characteristics of those involved in delivering Rounds was key to implementation success. Facilitators, specifically those that were seen to champion Rounds, were the main driving force behind implementation. Implementation worked best when they had strong support from an engaged clinical lead in a senior position (e.g., Head of School/Department), and from their steering group and administrator. There was diversity across sites in relation to how well these roles were filled (Table 6), with only two sites having all roles (Peony and Daisy), and one having none for the majority of the evaluation period (Daffodil, who were unable to implement Rounds). The influence of having/not having these deliverers and the collaboration between them are described in the following sections.

**Table 6:** Delivers of rounds across HEIs

Site	Facilitator champion	Senior leadership	Involved clinical lead	Supportive steering group	Admin support
Peony	✓	✓	✓	✓	✓
Daisy	✓	✓	✓	✓	✓
Cornflower	✓	✓	X	X	✓
Rose	X	✓	✓	✓	✓
Lavender	✓	X	✓	✓	X
Daffodil	X	X	X	X	X

#### Facilitators championing rounds

Implementation was most successful when one or more facilitators were observed to adopt the role to spearhead or ‘champion’ Rounds (Seen in four sites, Table 6). These facilitators were the driving force behind each aspect of implementation, including engaging with Schwartz South, proactively seeking support from senior leadership, organising the steering group and meetings, in addition to the day-to-day tasks associated with Rounds preparation. The long-term absence of the facilitator who initially championed Rounds at Daffodil was a key reason Rounds were not implemented, as there was no longer anyone leading the project, or able to obtain senior leadership support, nor appoint any of the additional roles.

Certain features were identified in those that championed Rounds, that subsequently aided implementation. For example, implementation was expedited if the champion had pre-existing experience and knowledge of Rounds. Peony and Daisy had facilitators that previously attended facilitation training in a healthcare practice organisation (e.g., NHS), so did not have to wait for training which was seen to cause delay at Rose. Having prior experience also gave facilitators more confidence in facing challenges to implementation. For example, when faced with low attendance in early Rounds, the facilitator at Daisy was observed to draw upon their prior experience to reassure steering group members. Confidence of facilitators provided a sense of capability which transferred to others involved in running Rounds:

*I might have a different opinion if I didn’t have such experienced facilitators… they know exactly what they’re doing… they’re perfectly capable of setting it up (Clinical Lead – Peony)*.

Passion for Rounds and their purpose was a common characteristic among the facilitators that led implementation. This passion was key in creating momentum by motivating both themselves and the steering group to drive implementation. In interviews, facilitators often stated how it was their interest and love for Rounds which encouraged them to be involved:

*In general*,* it’s one of the best things I do. I really do enjoy it ( Facilitator - Peony).*

However, these facilitators were observed to take on the majority of tasks involved in running Rounds, with other steering group members (and facilitators) relying on them to complete such tasks. Those championing Rounds noted the challenges of an increased workload of maintaining Rounds implementation, as allocated hours were not always sufficient:

*to put [Rounds facilitation] into a whole job as a lecturer where you have so many responsibilities and commitments*,* and this is an add-on*,* but it cannot take too much time (Facilitator – Daisy)*.

At Rose, although Rounds were implemented, there was no explicit champion, and therefore difficulty with gaining momentum was observed. For example, steering group meetings were well organised by the clinical lead and actions were set, but without a champion it appeared that there was a lack of ownership over Rounds and agreed actions were not always implemented once the steering group meeting had ended.

#### Seniority and involvement of clinical lead

In four of the five sites (Table 6), the role of clinical lead was fulfilled by a senior leader at their university. Clinical leads in senior positions provided oversight of implementation and support through having a direct line to the executive board in the HEI (responsible for allocating resource), which was useful in justifying Rounds, time commitment of staff members, and sourcing ongoing support:

*I think it’s not just about the money. It is about the support from leadership. It’s strategic leadership support (Clinical lead – Cornflower)*.

Those in senior positions had authority to schedule Rounds effectively around student timetables, selecting times when students were already on campus or avoiding placements and lectures (the most frequently reported enabler to attendance was the timing of the Round across all sites 33–100%). They were also able to leverage support from wider areas in the university such as marketing or web designers which facilitated successful delivery:

*Because I’m in a position to be able to action that quite quickly*,* and link people in with the marketing person or the website person*,* those actions happen quite quickly (Clinical lead – Peony)*.

Sites that did not have senior leadership either did not implement Rounds (Daffodil) or experienced delay (Lavender). In not having seniority, the clinical lead at Lavender faced challenges in setting up a steering group as it was more difficult to obtain permission for staff to meet and attend training or to recruit an administrator. Only with lobbying and additional work by the clinical lead (to cover administrative tasks and encourage staff members to join) was implementation successful.

The level of engagement of the clinical lead also influenced implementation. Engaged leads chased actions and set an example of involvement and engagement to the steering group. Leads who prioritised attending Rounds and steering group meetings provided motivation and feelings of support to the facilitators:

*And the fact that [clinical lead] is coming every time… so in the organisation*,* the head of department*,* it’s something he really loves coming to. It’s a nice feeling. (Facilitator – Daisy)*

When this wasn’t possible, for example when a lead had limited capacity, implementation momentum was seen to falter. At Cornflower, frequent non-attendance or rescheduling of steering group meetings by clinical leads appeared to reduce member engagement over time. Lower attendance and actions not followed up within and outside meetings was observed, leading to Rounds being cancelled.

#### Supportive steering group

Steering group involvement, whose role is to help plan and promote Rounds and source storytellers, was variable across HEIs. The most effective steering groups had active, enthusiastic members who would contribute within steering group meetings, take ownership of actions both within and beyond meetings and frequently talk to students about Rounds (e.g., engaging students within their own disciplines to increase attendance). Steering groups were most effective when shaped to the strengths of the members and the more effective they were, the more they reduced the demand on the facilitators and allowed for effective collaboration across all those delivering Rounds, which was considered essential for implementation success:

*We’ve sort of all been on the same page… we’re really like supportive of each other. We’ve got the same goal. I think that’s why it works so well (Facilitator -Lavender)*.

However, steering group involvement was an area of challenge for all sites. Facilitators at Cornflower and Rose discussed a lack of understanding of the expectations of the role of steering group member during observed meetings. It was thought that some steering group members viewed their role as only attending the meetings, but not activities beyond that:

*I think there lack of clarity at the very start about when you come into this steering group*,* what is it you’re being asked to do*,* and I think many people entered into it as… that sounds like a good idea*,* but not something that I’m necessarily going to commit my time towards*,* as in outside of these two hours or an hour in relation to my steering group. We have members of a steering group*,* for example*,* that have not attended a Schwartz Round (Facilitator - Rose)*.

Those with lower steering group involvement experienced greater challenges to implementation. For example, at Cornflower low attendance at steering group meetings hindered momentum for implementation as actions could not be agreed, previous Rounds could not be reviewed effectively, and future Rounds could not be planned. This added more burden and isolation onto the facilitator:

*you’re kind of just shouting into the ether … and nobody’s listening (Facilitator – Cornflower)*

A common difficulty across all sites was that steering group members were not allocated hours within their work plan for attending meetings, nor for additional activities required, and therefore other work-related responsibilities took priority:

*the start of our barriers [for steering group attendance] was the fact that… there was a staff survey that said nobody had time [for anything additional]*,* that everybody was being asked to do extra things (Steering group member – Lavender)*.

The importance of maintaining enthusiasm and reminding of responsibilities was noted across all sites. Even the most effective steering groups reported the need to refresh membership to accommodate changes in availability, passion and interest.

*I think you need to get people enthusiastic*,* get them on board and have an expectation of them*,* and if they don’t meet it*,* kindly let them go and find somebody that would like to do it (Administrator – Daisy)*.

#### Administrator support

Administrators played a fundamental role in supporting Rounds implementation by managing logistical responsibilities, allowing facilitators and steering group members more time to focus on other activities. However, their availability and involvement varied across sites. Two sites had no administrative support (Lavender and Daffodil). In the other four sites, their role varied. At Peony and Rose, administrators primarily fulfilled clerical duties as outlined by the PoCF guidance. In contrast, administrators at Daisy and Cornflower also adopted responsibilities of a steering group member such as helping name themes and promote Rounds by giving presentations in lectures.

There was no single model of administrative involvement that worked best; rather, successful implementation depended on how well the administration aligned with the needs of the delivery team. At Peony, a strong team of two facilitators and engaged clinical lead meant the clerical administrator role worked well. In Daisy and Cornflower, closer collaboration between administrator and facilitator was beneficial due to increased demands on a single facilitator and/or limited steering group involvement. Where administrators were integrated into the team, coordination was smoother and implementation more efficient. When there was poor fit, it left those running Rounds feel less supported:

*I think one thing that was really traumatic to me was that we got the first administrator wrong… And I really felt completely lonely the first round. (Facilitator – Daisy)*

At Lavender, the lack of administrative support posed a barrier. To address this, Rounds were scheduled within existing timetabled modules, which reduced logistical burden but may have impacted participation (explored below under *Student Engagement*).

### Inner setting: delivering rounds

Alongside ‘deliverers’, were key features about ‘delivering’ Rounds that influenced implementation success. When the deliverers (Rounds team) worked well together, these ‘delivering’ processes were more effective.

#### Student engagement

Engaging students to be aware of and attend Rounds was fundamental to implementation success across HEIs. This was done through Rounds promotion and student involvement.

#### Rounds promotion and ensuring clarity about what rounds are

Promoting Rounds to students was used to mitigate the barrier of students not knowing what Rounds were (18.1%, Table 5), or that they were being run at their HEI:

*the main difficulty that the team has right now is making sure that people attend the Rounds. And … a lot of times*,* it’s people knowing about them. (Administrator – Cornflower)*

Once students attended a Round, this encouraged future attendance. Survey data showed that 49.0% (0-56.4% across sites) of responders had been to at least one Round previously. As one student stated, they then knew what Rounds were and what they involved:

*I didn’t really know what was involved but today was very interesting. I will definitely attend the next one. (Attendee – Peony)*

As a result, promotional materials aimed to describe what Rounds were and provide the details for the upcoming Round. Promoting Rounds in lectures was deemed effective, particularly when delivered by a member of the Rounds team (deliverers) as they were authentic and engaging. In steering group meetings, members were observed discussing that they recognised students they had spoken to about Rounds in the audience. Student survey responses suggested that advertising Rounds using online or hardcopy posters was beneficial (23% of survey responses reported posters encouraged attendance, Table 5). However, it was noted that informal channels were the most effective. For example, students appeared most receptive when facilitators and steering group members frequently spoke to their students outside of lectures/seminars about Rounds:

*So*,* some of it and we’ve done a lot of really good formal things with promoting the Rounds*,* but on the most part*,* like all these things*,* quite often the informal channels are the ones that that just work. (Facilitator – Peony)*

Staff members who had good relationships with their students were perceived as having credibility when recommending Rounds, with some students noting that their tutor or lecturer personally encouraging them to attend was instrumental in their attendance.

In contrast, solely sending cohort wide emails with Rounds information was thought to be least effective:

*if we’re thinking I’m emailing out to 500-odd students*,* then we’re getting about 1–2% attendance. (Administrator - Rose)*

In interviews and steering group meetings, Schwartz team members from Peony and Cornflower discussed that students were likely to delete (or ignore) emails when they were unaware what Rounds were, and often were receiving multiple emails from the university.

As an incentive to attend, Cornflower trialled practice hours to be claimed by students attending Rounds, and Lavender timetabled Rounds within student modules. These strategies were both effective at increasing attendance. However, observational data suggested an unintended consequence whereby students in these sites were seen to be much less engaged in the stories and discussion, e.g. observed using their phones or having concurrent conversations with their friends.

#### Student involvement in implementation

To enhance student engagement, several sites partnered with students in implementation activities. At Peony, Lavender and Rose students were recruited as Schwartz representatives on steering groups based on the model used at Surrey. In observed steering group meetings, representatives provided student perspective on topics raised, shared peer feedback about how Rounds were received, and were asked to share communications with their peers. Students at Peony and Rose were also recruited to produce advertising materials, knowing what would engage students best.

Additionally, Peony and Daisy involved students by encouraging sign-up to Rounds through a dedicated social media site and mailing list respectively. Through these students received Round information, could provide feedback and were able to vote on polls regarding future theme choices. These strategies allowed the creation of a Schwartz community and helped tailor implementation to student needs, while providing students with ownership:

*It belongs to them, it’s for them. (Administrator – Daisy)*

#### Staff engagement

Raising Rounds awareness among staff members beyond the steering group increased the likelihood of them encouraging their students to attend and thereby increasing promotion to students. One strategy encouraged by the hub (Surrey) and used by three sites (Peony, Cornflower and Lavender) was to run separate staff Rounds and demonstrate firsthand what they involve and their benefit:

*(we wanted) to start with a staff round to get our faculty embedded and on side*,* and seeing the actual added benefits of that. (Facilitator – Cornflower)*

Through this, Peony also reported an increase in staff members expressing interest in joining the steering group, creating a waiting list of staff members to contact when a vacancy arose.

#### Embedding rounds into routine practice

During the study period, Peony, Daisy and Lavender demonstrated signs of embedding Rounds into routine university practice. In steering group meetings, focus shifted from early-stage concerns (e.g., increasing attendance, selling Rounds to the wider university, clarifying roles, and scheduling issues), to sustaining and refining Rounds. This was reflected in the activities undertaken by all Schwartz teams, who in early stages of implementation focused predominantly on informing students about what Rounds were. In those that began to embed Rounds, focus of actions shifted to planning and advertising multiple Rounds in advance, tailoring Rounds to specific events or student requests, encouraging more student contribution, and the creation and maintenance of additional resources (such as a webpage at Peony). Over the course of the study, in these sites, students were observed to demonstrate increasing familiarity with the structure and intention of Rounds, avoiding problem solving in Rounds and contributing relevant reflections, suggesting they were becoming ‘Schwartz savvy’ [17].

## Discussion

Investigation of the implementation of Schwartz Rounds within six HEIs has enabled the production of an evidence-based model describing the key drivers for successful implementation of Rounds in this setting. Successful implementation was enabled by the provision of financial and practical resources, and expertise provided by key players in the ‘external’ setting, namely the PoCF, the University of Surrey, and HEE. Following receipt of this support, it was the characteristics and inter-relationships of those leading and delivering Rounds alongside the engagement strategies they employed that impacted on success. Rounds are aimed at supporting health care students with the difficult, challenging work involved in care, giving space to reflect, learn and heal. Whilst these outcomes were not measured as part of this study, previous research would support that successful implementation would be associated with such outcomes [4, 19, 20].

While certain challenges were unique to HEI settings, several concurred with findings from previous evaluations of Rounds in healthcare settings. These included the burden of planning and running Rounds on facilitators, difficulties identifying optimal timing for Rounds, and the need to refresh steering group engagement over time [17]. As with previous Rounds evaluations [20], the effectiveness of drivers was not binary but operated as a dimmer switch. The greater their presence and strength, the more successfully Rounds were implemented and were then on a pathway to being embedded/sustained.

The implementation of interventions in HEIs, akin to most public sector organisations, is often dependent on financial considerations. At present many HEIs in the UK face financial pressures [32, 33], making the cost of new initiatives a potential barrier to implementation. In this study, external financial support from Schwartz South (via HEE) that covered licencing, training, food and admin costs, likely facilitated organisational commitment and support for Rounds. Similar schemes/projects for other interventions have similarly highlighted the importance of funding in initiating such programmes [34, 35]. Despite this support, financial sustainability remained a concern beyond the study period. Some sites addressed this by developing internal business cases, often led by engaged, senior leads to secure long-term institutional support and to cover staff time. Nonetheless, securing consistent internal funding remains a recognised barrier to the sustained implementation of Schwartz Rounds in HEIs [24].

Finance alone was not sufficient for successful implementation. Our findings concurred with previous research highlighting the importance of having strong, passionate leaders, those that champion Rounds, and a wider supportive Schwartz team [17, 36]. The pivotal role of champions in implementing interventions within healthcare and HEI settings is well-documented. Champions have been shown to facilitate the adoption of evidence-based practices, helping overcome barriers and fostering engagement [37, 38]. However, these individuals often face demands that add to already heavy workloads [17, 36, 37] or exceed allocated hours as seen in the current study. Reliance on a small group of committed individuals raises sustainability concerns, particularly within the context of limited financial resource in HEIs. Issues may arise when staff leave or are unavailable. In sites that embedded Rounds over this longitudinal study, they had senior leader support and an effective steering group who shared demands more effectively and thereby may act as buffers if key champions leave.

Engagement and attendance challenges also replicated those reported in previous studies of Rounds in healthcare settings [17, 29]. Lack of awareness and understanding about Rounds remained a key barrier despite the various materials developed to try to mitigate this (e.g. films [39, 40], posters). Consistent with prior research, tailored promotion strategies and well-chosen session times (albeit no optimal time) were shown to support student participation [24]. In addition to endorsement in lectures by genuine, trusted staff (e.g., tutors from steering group), involving students in implementation was an effective engagement strategy. Giving students ownership of the intervention supported its integration into student culture at the HEI. This supports evidence of highlighting the value of students as active partners in decision-making and co-design in higher education initiatives, which enhances relevance, awareness and uptake [41, 42]. Additionally obtaining ‘buy-in’ from wider university staff who can help deliver information or engagement strategies of interventions was effective [43].

Aligning with Normalisation Process Theory [27] sites started to embed Rounds within their university processes over the study period resulting in them going beyond ‘first order’ improvements toward systemic change. The longitudinal design of this study allowed the observation of initial implementation efforts to early stages of integration within institutional culture, underscoring that successful implementation is not a discrete event but an ongoing, adaptive process [44]. This process involves multiple outcomes, including immediate attendance and engagement as well as longer-term shifts in organisational culture, such as fostering a more compassionate, reflective environment. Culture change in such contexts is typically gradual, occurring through small, incremental steps rather than rapid transformation [45] as also evidenced in the national evaluation of Rounds in the NHS [17].

### Strengths and limitations

This study was underpinned by the robust evidence base arising from previous evaluations of Schwartz Rounds [17] and comparable interventions (i.e., Team Time [29]). It was unique in its inclusion of multiple sites, enabling cross-case analysis, allowing for the identification of common and context-specific drivers of implementation. The longitudinal design enabled examination of the implementation process and offered insights into how Rounds could become embedded over time. Additionally, the integration of data from multiple methods and sources (interview, observations and surveys) facilitated methodological triangulation and enhanced the credibility of findings by capturing diverse perspectives and considering the context at each HEI.

The study is limited by challenges in data collection leading to inconsistencies in the data available across the sites. We were unable to interview ‘non’ attendees, so interview data is likely to be skewed to be from those more engaged in Rounds. There was also wide variability in relation to survey response rates between the sites, meaning findings that used this data are skewed to sites that ran more Rounds and achieved higher response rates. The mixed methods design, and triangulation of interview, observation and survey data helped to mitigate these limitations. Finally, the study period was not long enough to assess factors influencing the sustainability of Schwartz Rounds beyond the implementation phase. Future research should look to explore sustainability of Rounds within HEIs.

## Conclusion

The findings of this study offer new insight into how Rounds are implemented in HEIs and the enablers and barriers experienced. Successful implementation, defined in this study as delivery with high fidelity to the PoCF model, sustained uptake and acceptability, and integration into organisational strategy and practices, was influenced by local adaption, leadership engagement, team support and student involvement. Where this happened, there was stronger evidence of positive impact on students learning and wellbeing/feeling of support.

While HEIs present distinct contextual challenges, many implementation issues mirrored those reported in healthcare settings, including reliance on committed facilitators, challenges around timing and attendance, and the need for sustained steering group engagement. Importantly, early signs of embedding were observed where Rounds became increasingly familiar to students and integrated into institutional processes, suggesting potential for longer-term impact within HEIs.

These findings offer practical guidance for HEIs seeking to implement Schwartz Rounds and support student wellbeing, highlighting the importance of investment not only in financial and external support, but also in leadership, team capacity, and partnership with students. More broadly, this study contributes to the growing evidence base on implementing wellbeing-focused interventions in educational settings and underscores that successful implementation is an ongoing, relational process rather than a discrete event.

## Supplementary Information

Supplementary Material 1.

## Data Availability

The datasets generated and/or analysed during the current study are not publicly available due to the need to ensure they are appropriately anonymised but are available from the corresponding author on reasonable request.

## Abbreviations

CFIR: Consolidated Framework of Implementation Research
HEE: Health Education England
HEI: Higher Education Institution
NHS: National Health Service
PoCF: Point of Care Foundation

## References

[1] World Health Organization. State of the world’s nursing 2020: investing in education, jobs and leadership. Geneva: World Health Organization; 2020. Available from: https://www.who.int/publications/i/item/9789240003279. Accessed 27 Jun 2025.

[2] World Health Organization. Health and care workforce in Europe: time to act. Copenhagen: WHO Regional Office for Europe; 2022. Available from: https://www.who.int/europe/publications/i/item/9789289058339. Accessed 27 Jun 2025.

[3] Asturias N, Andrew S, Boardman G, Kerr D. The influence of socio-demographic factors on stress and coping strategies among undergraduate nursing students. Nurse Educ Today. 2021;99:104780. 33516979 10.1016/j.nedt.2021.104780

[4] Hamilton D, Taylor C, Maben J. How does a group reflection intervention (Schwartz rounds) work within healthcare undergraduate settings? A realist review. Perspect Med Educ. 2023;12(1):550. 38144671 10.5334/pme.930 PMC10742148

[5] Canzan F, Saiani L, Mezzalira E, Allegrini E, Caliaro A, Ambrosi E. Why do nursing students leave bachelor program? Findings from a qualitative descriptive study. BMC Nurs. 2022;21(1):71. 35351118 10.1186/s12912-022-00851-z PMC8966353

[6] Pulido-Martos M, Augusto‐Landa JM, Lopez‐Zafra E. Sources of stress in nursing students: a systematic review of quantitative studies. Int Nurs Rev. 2012;59(1):15–25.

[7] Michinov E, Robin G, Hémon B, Béranger R, Boissart M. Protective resources against stress among student nurses: influences of self-efficacy, emotional intelligence and conflict management styles. Nurse Educ Pract. 2024;74:103849. 38006646 10.1016/j.nepr.2023.103849

[8] Soler OM, Aguayo-González M, Gutiérrez SS, Pera MJ, Leyva-Moral JM. Nursing students’ expectations of their first clinical placement: A qualitative study. Nurse Educ Today. 2021;98:104736. 33493924 10.1016/j.nedt.2020.104736

[9] Rowland E, Trueman H. Improving healthcare student experience of clinical placements. BMJ Open Qual; 2024;13(1):1–9. 10.1136/bmjoq-2023-002504 PMC10773407 38176708

[10] Jack KF. The meaning of compassion fatigue to student nurses: an interpretive phenomenological study. J Compassionate Health Care. 2017;4:2.

[11] Maben J, Adams M, Peccei R, Murrells T, Robert G. Poppets and parcels’: the links between staff experience of work and acutely ill older peoples’ experience of hospital care. Int J Older People Nurs. 2012;7(2):83–94. 22531048 10.1111/j.1748-3743.2012.00326.x

[12] Vatansever N, Akansel N. Intensive care unit experience of nursing students during their clinical placements: a qualitative study. Int J Caring Sci. 2016;9(3):1040–8.

[13] British Medical Association. ‘Performative, not practised’ – wellbeing provisions for UK medical students fall short, warns BMA. London: British Medical Association; 2024. Available from: https://www.bma.org.uk/news-and-opinion/performative-not-practised-wellbeing-provisions-for-uk-medical-students. Accessed 27 Jun 2025.

[14] Edge D, Gladstone N. Exploring support strategies for improving nursing student retention. Nurs Standard. 2022;37(9):28. 10.7748/ns.2022.e11914 35912439

[15] Health Education England. Reducing pre-registration attrition and improving retention. 2020. Available from: https://www.hee.nhs.uk/our-work/reducing-pre-registration-attrition-improving-retention. Accessed 27 Jun 2025.

[16] Schwartz K. A patient’s story. Boston Globe Magazine. Boston: Boston Globe Magazine; 1995. Available from: https://www.bostonglobe.com/magazine/1995/07/16/patient-story/q8ihHg8LfyinPA25Tg5JRN/story.html. Accessed 27 Jun 2025.

[17] Maben J, Taylor C, Dawson J, Leamy MC, McCarthy I, Reynolds EF, et al. A realist informed mixed-methods evaluation of Schwartz Center Rounds^®^ in England. Health Serv Deliv Res. 2018;6(37):1–260.

[18] Point of Care Foundation. Schwartz Rounds information pack for larger organisations . London: The Point of Care Foundation; 2017. Available from: https://www.pointofcarefoundation.org.uk/wp-content/uploads/2017/01/Attach-1.-New-Information-pack-big.pdf. Accessed 27 Oct 2025.

[19] Taylor C, Xyrichis A, Leamy MC, Reynolds E, Maben J. Can Schwartz center rounds support healthcare staff with emotional challenges at work, and how do they compare with other interventions aimed at providing similar support? A systematic review and scoping reviews. BMJ Open. 2018;8(10):e024254. 10.1136/bmjopen-2018-024254 PMC6196967 30341142

[20] Maben J, Taylor C, Reynolds E, McCarthy I, Leamy M. Realist evaluation of Schwartz rounds^®^ for enhancing the delivery of compassionate healthcare: Understanding how they work, for whom, and in what contexts. BMC Health Serv Res. 2021;21:1–24. 34275468 10.1186/s12913-021-06483-4 PMC8286624

[21] Corless IB, Michel TH, Nicholas M, Nokes KM, Sefcik EF. Educating health professions students about the issues involved in communicating effectively: a novel approach. J Nurs Educ. 2009;48(7):367–73. 19634261 10.3928/01484834-20090615-03

[22] Gishen F, Whitman S, Gill D, Barker R, Walker S. Schwartz centre rounds: a new initiative in the undergraduate curriculum—what do medical students think? BMC Med Educ. 2016;16:1–7. 27658411 10.1186/s12909-016-0762-6 PMC5034622

[23] Stocker C, Cooney A, Thomas P, Kumaravel B, Langlands K, Hearn J. Schwartz rounds in undergraduate medical education facilitates active reflection and individual identification of learning need. MedEdPublish. 2018;7:230. 38089201 10.15694/mep.2018.0000230.1 PMC10712006

[24] Zile A, Owen J, Gorick H, Orford A, Panagiotaki G. Schwartz rounds in higher education settings: A systematic review of the research with recommendations. J Med Educ Curric Dev. 2025;12:23821205251320152. 40008116 10.1177/23821205251320152 PMC11851745

[25] Grimbly V, Golding L. Running interprofessional Schwartz Rounds with healthcare students in the North of England: Building capacity and evaluating impact. Liverpool: University of Liverpool; 2023. Available from: https://www.liverpool.ac.uk/media/livacuk/iphs/psychologicalsciences/2019-2022,Final,Schwartz,North,Report-310123.pdf. Accessed 10 Oct 25.

[26] University of Surrey. Schwartz South project. Guildford: University of Surrey; 2022. Available from: https://www.surrey.ac.uk/research-projects/schwartz-south. Accessed 27 Jun 2025.

[27] May C, Finch T. Implementing, embedding, and integrating practices: an outline of normalization process theory. Sociology. 2009;43(3):535–54.

[28] Student Schwartz Rounds – University of Surrey. Workforce Research Surrey. Available from: https://workforceresearchsurrey.health/schwartz-rounds/university-schwartz-rounds/. Accessed 10 Oct 2025.

[29] Zasada M, Van Even S, Maben J, Oates J, Taylor C. Team Time as a wellbeing intervention for NHS staff: a qualitative evaluation of implementation during the COVID-19 pandemic. BMC Health Services Research. 2024;24(1):1622.Gale 2013. 10.1186/s12913-024-12046-0 PMC11657638 39695659

[30] Gale NK, Heath G, Cameron E, Rashid S, Redwood S. Using the framework method for the analysis of qualitative data in multi-disciplinary health research. BMC Med Res Methodol. 2013;13(1):117. 24047204 10.1186/1471-2288-13-117 PMC3848812

[31] Damschroder LJ, Reardon CM, Widerquist MA, Lowery J. The updated consolidated framework for implementation research based on user feedback. Implement Sci. 2022;17(1):75. 36309746 10.1186/s13012-022-01245-0 PMC9617234

[32] Lewis J, Bolton P. Higher education funding: trends and challenges. London: House of Commons Library; 2024. Available from: https://commonslibrary.parliament.uk/higher-education-funding-trends-and-challenges/. Accessed 16 Jul 2025.

[33] Universities UK. Universities grip financial crisis – but at what cost to the nation? London: Universities UK; 2025 [updated 2025]. Available from: https://www.universitiesuk.ac.uk/what-we-do/creating-voice-our-members/media-releases/universities-grip-financial-crisis-what. Accessed 27 Jun 2025.

[34] Maillé S, Beaulieu F, Lachance L, Grégoire S. Barriers and facilitators to the implementation of a peer support intervention in universities. J Coll Student Mental Health. 2025;39(1):86–109.

[35] Robert G, Philippou J, Leamy M, Reynolds E, Ross S, Bennett L, Taylor C, Shuldham C, Maben J. Exploring the adoption of Schwartz center rounds as an organisational innovation to improve staff well-being in England, 2009–2015. BMJ Open. 2017;7(1):e014326. 10.1136/bmjopen-2016-014326 PMC5223680 28057662

[36] Wilkins D, Thompson S, Jones R, Bezeczky Z, Bennett V. Implementing Schwartz rounds in children’s social care: enablers and barriers. J Social Work. 2025;25(1):61–82.

[37] Farr M, Barker R. Can staff be supported to deliver compassionate care through implementing Schwartz rounds in community and mental health services? Qual Health Res. 2017;27(11):1652–63. 28799475 10.1177/1049732317702101

[38] Morena AL, Gaias LM, Larkin C. Understanding the role of clinical champions and their impact on clinician behavior change: the need for causal pathway mechanisms. Front Health Serv. 2022;2:896885. 36925794 10.3389/frhs.2022.896885 PMC10012807

[39] Health Workforce Research - University of Surrey. University Schwartz Rounds: supporting students. YouTube. 2024. Available from: https://www.youtube.com/watch?v=GvmItJ3PIKQ. Accessed 01 Aug 2025.

[40] Health Workforce Research - University of Surrey. Schwartz Rounds: supporting new staff. YouTube. 2024 February 29. Available from: https://www.youtube.com/watch?v=GvmItJ3PIKQ. Accessed 01 Aug 2025.

[41] Bovill C. An investigation of co-created curricula within higher education in the UK, Ireland and the USA. Innovations Educ Teach Int. 2014;51(1):15–25.

[42] Cook-Sather A, Bovill C, Felten P. Engaging students as partners in learning and teaching: a guide for faculty. San Francisco: Jossey-Bass; 2014.

[43] Crabtree RM, Briggs P, Woratschek H. Student engagement and barriers to implementation: the view of professional and academic staff. Perspectives: Policy Pract High Educ. 2021;25(4):144–50.

[44] Greenhalgh T, Wherton J, Papoutsi C, Lynch J, Hughes G, A’Court C, et al. Beyond adoption: a new framework for theorizing and evaluating nonadoption, abandonment, and challenges to the scale-up, spread, and sustainability of health and care technologies. J Med Internet Res. 2017;19(11):e367. 29092808 10.2196/jmir.8775 PMC5688245

[45] Bate P, Robert G, Bevan H. The next phase of healthcare improvement: what can we learn from social movements? BMJ Qual Saf. 2004;13(1):62–6. 10.1136/qshc.2003.006965 PMC1758052 14757802

