# Supplementary material for: Exploring factors that contribute to the successful implementation of Schwartz Rounds in higher education institutions
Source: BMC Med Educ. 2026 Feb 11;26:427. doi: 10.1186/s12909-026-08793-9 (PMC12998277; doi:10.1186/s12909-026-08793-9)
Supplement: Supplementary file 1 — Supplementary Material 1. [file 12909_2026_8793_MOESM1_ESM.docx]

Supplementary Table 1: All enablers and barriers to student attendance reported in survey

|  | Peony % (N) | Daisy  % (N) | Cornflower  % (N) | Rose % (N) | Lavender  % (N)^ | Total  % (N) |
| --- | --- | --- | --- | --- | --- | --- |
| Total N Responses: | 318 | 34 | 14 | 2 | 113 | 481 |
| What encouraged you to attend this Round? |  |  |  |  |  |  |
| It was online and easy to attend | 30.8 (98) | N/A | 28.5 (4) | 100.0 (2) | N/A | 21.6 (104) |
| The time of day was right | 55.7 (177) | 73.5 (25) | 57.1 (8) | 100.0 (2) | 32.7 (37) | 51.8 (249) |
| I was not in placement/clinical practice | 36.5 (116) | 41.2 (14) | 28.5 (4) | 0.0 (0) | 22.1 (25) | 33.1 (159) |
| I was given time in placement/clinical practice/ during class | 16.7 (53) | 23.5 (8) | 0.0 (0) | 50.0 (1) | 17.7 (20) | 17.0 (82) |
| It was face-to-face and I was already in college | 24.2 (77) | 47.1 (16) | 0.0 (0) | N/A | 49.6 (56) | 30.1 (149) |
| I saw a poster and the topic appealed to me | 30.5 (97) | 8.8 (3) | 21.4 (3) | 50.0 (1) | 3.5 (4) | 22.5 (108) |
| My friend suggested I attend/ I went with a friend | 24.5 (78) | 11.8 (4) | 7.1 (1) | 0.0 (0) | 2.7 (3) | 17.9 (86) |
| I knew the storyteller | 7.2 (23) | 0.0 (0) | 7.1 (1) | 0.0 (0) | 4.4 (5) | 6.0 (29) |
| The storyteller was from my field of practice | 13.8 (44) | 11.8 (4) | 7.1 (1) | 0.0 (0) | 10.6 (12) | 12.7 (61) |
| Attended a previous Round* | 4.7 (15) | 2.9 (1) | 0.0 (0) | 0.0 (0) | 0.0 (0) | 3.3 (16) |
| Promoted by lecturers * | 5.7 (18) | 8.7 (3) | 14.3 (2) | 0.0 (0) | 1.8 (2) | 6.2 (25) |
| Intrigued/ curious by experience/ liked sound of it | 2.8 (9) | 0.0 (0) | 0 (0.0) | 0.0 (0) | 0.9 (1) | 2.1 (10) |
| Heard good things (peers)* | 0.6 (2) | 0.0 (0) | 0 (0.0) | 0.0 (0) | 0.0 (0) | 0.4 (2) |
| Round was timetabled or practice hours* | 1.3 (4) | 0.0 (0) | 0 (0.0) | 0.0 (0) | 7.1 (8) | 2.5 (12) |
| Food* | 0.3 (1) | 0.0 (0) | 0 (0.0) | 0.0 (0) | 0.0 (0) | 0.2(1) |
| Are there any challenges you experience in attending Rounds? |  |  |  |  |  |  |
| Difficult to spare the time | 23.9 (76) | 26.5 (9) | 35.7 (5) | 0.0 (0) | 8.0 (9) | 20.6 (99) |
| I didn’t know what was involved | 14.5 (46) | 32.4 (11) | 7.1 (1) | 0.0 (0) | 25.7 (29) | 18.1 (87) |
| I couldn’t get to college for the Round (face-to-face) | 11.9 (38) | 23.5 (8) | 7.1 (1) | N/A | 1.8 (2) | 10.2 (49) |
| Too expensive to get to college for the Round (face-to-face) | 5.3 (17) | 11.8 (4) | 7.1 (1) | 50.0 (1) | 1.8 (2) | 5.2 (25) |
| Rounds are scheduled at a bad time of day for me | 9.4 (30) | 23.5 (8) | 14.3 (2) | 50.0 (1) | 3.5 (4) | 9.4 (45) |
| Previous topics didn’t appeal to me | 1.3 (4) | 0.0 (0) | 0.0 (0) | 0.0 (0) | 1.8 (2) | 1.2 (6) |
| Couldn’t get time during placement to attend* | 1.3 (4) | 2.9 (1) | 0.0 (0) | 0.0 (0) | 0.0 (0) | 1.0 (5) |
| Wasn’t aware of Rounds being held* | 4.1 (13) | 2.9 (1) | 7.1 (1) | 0.0 (0) | 0.9 (1) | 3.3 (16) |
| Accessibility (Neurodivergent, physical discomfort) * | 0.6 (2) | 0.0 (0) | 0.0 (0) | 0.0 (0) | 0.0 (0) | 0.4 (2) |
| Prefer in person events* | 1.3 (4) | 0.0 (0) | 0.0 (0) | 0.0 (0) | 0.0 (0) | 0.8 (4) |
| Discomfort in sharing story/ fear of speaking* | 0.3 (1) | 2.9 (1) | 0.0 (0) | 0.0 (0) | 0.9 (1) | 0.6 (3) |
| No barriers* | 4.4 (14) | 5.9 (2) | 7.1 (1) | 0.0 (0) | 9.7 (11) | 5.8 (28) |

** Indicates new field based on content analysis of free text; Note that attendees could select more than one option; All Daisy and Lavender Rounds were in face-to-face, all Rose Rounds were online; ^Rounds were timetabled within modules for students;*

Supplementary Table 2: Key drivers mapped to the CFIR

| Key Drivers | CFIR Domain | CFIR Construct |
| --- | --- | --- |
| External Organisations | Outer setting | Financing, partnerships,  Innovation source, evidence base |
| Deliverers of Rounds | Inner setting  Individuals (Roles & Characteristics) |  |
| - Facilitators championing Rounds |  | Implementation leads, capability, motivation |
| - Seniority and involvement of clinical lead |  | High-level leaders, opinion leaders, relative priority, available resources |
| - Supportive steering group |  | Implementation team members, teaming, opportunity, planning |
| - Administrator support |  | Implementation team members, teaming, planning |
| Delivering Rounds | Inner setting  Implementation process |  |
| - Student engagement |  | Engaging (innovation recipients and deliverers) assessing needs, tailoring strategies |
| - Staff engagement |  | Engaging (innovation deliverers) |
| - Embedding Rounds into routine practice |  | Adapting, Reflecting |

Supplementary materials

Supplementary material 1: Post-Rounds attendance questionnaire

1. Please tick the response that most reflects your opinion of today’s Schwartz Round.

| **Statement** | Completely disagree | Disagree somewhat | Neither agree or disagree | Agree somewhat | Completely agree |
| --- | --- | --- | --- | --- | --- |
| The Schwartz Round provided a space that felt distinct from other university/ work related meetings and spaces (e.g. allowed me to put myself first. Not feel assessed or problem solve) |  |  |  |  |  |
| It felt safe to share my experiences and feelings, I did not feel that I would be judged if I had shared my experiences |  |  |  |  |  |
| Hearing others share difficult, challenging and/or satisfying experiences about their work makes it easier for me to share my experiences |  |  |  |  |  |
| Hearing the storytellers’ stories today has enabled me to learn about experiences and roles that I did not fully appreciate before |  |  |  |  |  |
| Hearing the storytellers’ stories today has enabled me a greater insight into the work undertaken by other students and colleagues in practice, to see beyond their ‘job title’ and increase the empathy I feel towards them |  |  |  |  |  |
| Hearing storytellers’ stories today has enabled greater insight into patients/relatives/students’ lives, and will change the way I see them |  |  |  |  |  |
| Attending todays Round enabled me to reflect on my role and experiences |  |  |  |  |  |
| I feel less alone in my experiences and that others felt the same as me |  |  |  |  |  |
| I gained knowledge that will help me to care for my patients/ relatives and/or others I study/ work with |  |  |  |  |  |
| The group discussion was well facilitated |  |  |  |  |  |
| I plan to attend Schwartz Rounds again |  |  |  |  |  |
| I would recommend Schwartz Rounds to colleagues |  |  |  |  |  |

1. Please rate today’s Round (circle as appropriate)

| Exceptional | | Excellent | | Good | | Fair | | Poor |
| --- | --- | --- | --- | --- | --- | --- | --- | --- |
|  |  | |  | |  | |  |  |

1. What encouraged you to attend this Schwartz Round? Please tick all that apply

| It was online and easy to attend |  |
| --- | --- |
| Time of day was right |  |
| Not in placement/clinical practice |  |
| Given time in placement/, clinical practice/ during class |  |
| Was face-to-face and I was already in college |  |
| Saw a poster and the topic appealed to me |  |
| My friend suggested I attend/ I went with a friend |  |
| I knew the storyteller |  |
| The storyteller was from my field of practice/study |  |
| Other (please specify) | |

1. Please could you share with us any comments or feedback you have about this latest Schwartz Round? (What worked well, any suggestions for improvement?)
2. Did you speak in the Round today? (circle as appropriate)

Yes No I didn’t want to No but I wanted to

**If YES**: what made you feel safe to do this?

**If NO BUT I WANTED TO**: was there a reason for this?

1. Were there educators/teachers present at the Round today? (please circle)

Yes No I don’t know

**If NO:** Please skip to question 7.

**If YES**: Did that impact (either positively or negatively) on your contribution?

Yes No

Please explain your answer

1. How many Rounds have you attended previously?

0 1-2 3-5 More than 5 More than 10
Other (please specify) ________

1. Are there any barriers/challenges you experience in attending Rounds? (tick all that apply)

| Difficult to spare the time |  |
| --- | --- |
| I didn’t know what was involved |  |
| I couldn’t get into college for the Round (face-to-face) |  |
| Too expensive to get into college for the Round (face-to-face) |  |
| Rounds are scheduled at a bad time of day for me |  |
| Previous topics didn’t appeal to me |  |
| Other (please specify) | |

1. **IF POST-REGISTRATION:** What encourages you to keep attending Rounds after graduating?

**IF IN YEAR 1, 2, 3 OR FOUR:** What would encourage you to keep taking part in Rounds after you graduate?

1. Do you have any comments on how Rounds have impacted you? (For example you thought about them afterwards/ shared with friends/colleagues/ changed your practice/ changed your behaviour as a result of Rounds?)
2. Do you have any theme suggestions for future Rounds?

Supplementary material 2: Non-participant observation form

**Schwartz Round observation V01 03/10/2022**

| **Site:** |  | **No. facilitators** |  |
| --- | --- | --- | --- |
| **Team:** |  | **No. storytellers:** |  |
| **Day & date:** |  | **Others present (steering group, observers etc.)** |  |
| **Time starts:** |  |  |  |

**Session timings**

| **Room open/ Invited in** |  |
| --- | --- |
| **Introduction** |  |
| **Story 1** |  |
| **Story 2** |  |
| **Story 3** |  |
| **Audience reflections/discussion** |  |
| **Closing comments** |  |
| **End** |  |
| **Other** |  |

**Attendance and participation**

(If online) Room locked at start of session

| **Time into session** | **No. of audience members present (approx. -30 max)** | **Tally of speaking audience members:** | **Tally of audience members contributing to online chat:** |
| --- | --- | --- | --- |
|  |  |  |  |
|  |  |  |  |
|  |  |  |  |
|  |  |  |  |

Approx. total **__________** Approx. total **_________**

**Storytellers:**

| **Gender** | **Professional group** | **Same team as the audience?** |
| --- | --- | --- |
|  |  |  |
|  |  |  |
|  |  |  |

*If more than one storyteller*

Were they prepared together?  Yes  No  Not sure

Were they in one room together?  Yes  No

**Facilitator roles** *(tick as appropriate)*

| **Elements of facilitator role** | **Facilitator 1** | **Facilitator 2** | **Comments** |
| --- | --- | --- | --- |
| Introduction to Schwartz Round session |  |  |  |
| Introduction of storytellers |  |  |  |
| Start of discussion |  |  |  |
| Managing/contributing to discussion |  |  |  |
| End of session |  |  |  |
| Other (specify) |  |  |  |

**THEME/TITLE: _______________________________________________________________**

**Facilitation according to PoCF protocol** *(add any comments relating to the below)*

- Setting boundaries for storyteller and audience participants safety (e.g. handling emotions, managing blame, nothing political/inappropriate is being discussed)
- Creating a space that is sensitive to the individual speaking, their work role, and the needs of the group
- Time management: explicit about how time will be managed, managing time equitably throughout, balancing contributions from the participants and the storytellers, balancing questions and reflections, ensuring all have time to speak
- Managing group processes effectively to create opportunities for silence and speaking, attending to the range of emotions in the group.
- Redirecting to avoid problem solving and too much clinical detail
- Drawing out the meaning underlying the staff and patient stories, summarising themes, and posing questions to storytellers and participants, to encourage reflection. Backup stories/reflection?
- Maintaining a position of neutral curiosity

**Other active team roles in running session i.e., tech support, steering group members:**

**Any issues experienced in the Round? (e.g. no discussion, disagreement, room problem?)**

**If online: Any technical issues (connection, sound, video): who? when?**

**Session start**

Food and space/time to unwind

If online: Waiting room

**Introduction** – If online: Audience cameras  on  off  partial

Introduction by Facilitators *tick all that apply*

What Schwartz Rounds are (reflection and group discussion)

How they works (incl. role of participants)

Structure of session (incl. timings)

Confidentiality statement –agreed individually? ____________________________

Rounds etiquette (e.g., no interrupting) – (If online: e.g., indication of when cameras on or off, switching off mobile phones and bleepers)

Silence

Not problem solving – key aim is reflection

Introduction of facilitators, and observers

Grounding techniques (e.g., mindful breathing techniques and butterfly hug – for all participants)

Discussion – explained to participants how to participate by raising their hand or if online by writing in the ‘chat’ function or clicking on the ‘hand-up’ emoji

Making sure that the group knows that they will get back to work promptly

☐ Completing questionnaire before leaving

☐ What today's topic is

**Presentation of story** – If online: Audience cameras  on  off  partial

If in person: Audience engaged ?  Yes  No  Partially

**Please describe key event(s)/experience(s)/emotion(s) below**

Storyteller 1

☐ Introduced themselves at start of story

Storyteller 2

☐ Introduced themselves at start of story

Storyteller 3

☐ Introduced themselves at start of story

Do the Facilitators stick to the “take home message” as a full stop after the storyteller(s) has finished their story? [to “close” the story and prevent “what happened next” questions]  Yes  No

**Audience reflection/ discussion**

If online: Audience cameras  on  off  partial

Is chat function used? How?

In person: How did people show they were willing to share their thoughts?

Audience contributions: What are they contributing (deepening of the story, own stories, comments on what resonated with them)? Are any questions asked? How does the discussion develop? What are explicit and implicit messages being conveyed?

**Management of emotions and interpersonal dynamics**

Audience support for each other / storytellers? Are there obvious displays of emotion? How do the facilitators/participants respond (please list any verbal/non-verbal reactions below)?

Other observations relating to atmosphere and dynamics e.g., key moments, silences, more than one person speaking at once, turn taking, any humour etc

**End of Round** – If online: Audience cameras  on  off  partial

Do the facilitators summarise some themes that have arisen?  Yes  No

Do the facilitators thank the storytellers? ☐ Yes ☐ No

Do the facilitators ask if the storytellers want to make any final comments? ☐ Yes ☐ No

Thoughts from storytellers:

Is a link for evaluation forms provided? ☐ Yes ☐ No

How?_____________________________

Are participants signposted for further support if needed?

Do the facilitator(s) share a date of the next Schwartz Round? ☐ Yes ☐ No

Do the facilitator(s) invite participants to offer topics for future sessions, directing them to the intranet page, or offering other ways to contact the facilitator(s) or the steering group with suggestions? ☐ Yes ☐ No

Observations about how Schwartz Round session ends: Do they stay in the space after for social reasons? What happens at the end? Do they debrief with the storyteller(s)? How/where?

**Any other observations:**
